# Determination of antibacterial activity and minimum inhibitory concentration of larval extract of fly via resazurin-based turbidometric assay

**DOI:** 10.1186/s12866-017-0936-3

**Published:** 2017-02-16

**Authors:** Chien Huey Teh, Wasi Ahmad Nazni, Ab Hamid Nurulhusna, Ahmad Norazah, Han Lim Lee

**Affiliations:** Medical Entomology Unit, Infectious Disease Research Centre, Institute for Medical Research, Jalan Pahang, Kuala Lumpur, 50588 Malaysia

**Keywords:** *Lucilia cuprina*, Maggot therapy, Antibacterial activity, Resazurin, MRSA

## Abstract

**Background:**

Antimicrobial resistance is currently a major global issue. As the rate of emergence of antimicrobial resistance has superseded the rate of discovery and introduction of new effective drugs, the medical arsenal now is experiencing shortage of effective drugs to combat diseases, particularly against diseases caused by the dreadful multidrug-resistant strains, such as the methicillin-resistant *Staphylococcus aureus* (MRSA). The ability of fly larvae to thrive in septic habitats has prompted us to determine the antibacterial activity and minimum inhibitory concentrations (MICs) of larval extract of flies, namely *Lucilia cuprina, Sarcophaga peregrina* and *Musca domestica* against 4 pathogenic bacteria [*Staphylococcus aureus,* methicillin-resistant *S. aureus* (MRSA), *Pseudomonas aeruginosa* and *Escherichia coli*] via a simple and sensitive antibacterial assay, resazurin-based turbidometric (TB) assay as well as to demonstrate the preliminary chemical profile of larval extracts using gas chromatography-mass spectrophotometry (GC-MS).

**Results:**

The resazurin-based TB assay demonstrated that the *L. cuprina* larval extract was inhibitory against all tested bacteria, whilst the larval extract of *S. peregrina* and *M. domestica* were only inhibitory against the MRSA, with a MIC of 100 mg ml^-1^. Subsequent sub-culture of aliquots revealed that the larval extract of *L. cuprina* was bactericidal against MRSA whilst the larval extracts of *S. peregrina* and *M. domestica* were bacteriostatic against MRSA. The GC-MS analysis had quantitatively identified 20 organic compounds (fatty acids or their derivatives, aromatic acid esters, glycosides and phenol) from the larval extract of *L. cuprina*; and 5 fatty acid derivatives with known antimicrobial activities from *S. peregrina* and *M. domestica*.

**Conclusion:**

The resazurin-based turbidometric assay is a simple, reliable and feasible screening assay which evidently demonstrated the antibacterial activity of all fly larval extracts, primarily against the MRSA. The larval extract of *L. cuprina* exerted a broad spectrum antibacterial activity against all tested bacteria. The present study revealed probable development and use of novel and effective natural disinfectant(s) and antibacterial agent(s) from flies and efforts to screen more fly species for antibacterial activity using resazurin-based TB assay should be undertaken for initial screening for subsequent discovery and isolation of potential novel antimicrobial substances, particularly against the multi-drug resistant strains.

**Electronic supplementary material:**

The online version of this article (doi:10.1186/s12866-017-0936-3) contains supplementary material, which is available to authorized users.

## Background

The advent of antibiotics had improved mankind’s health status and quality of life tremendously. However, overuse and misuse of antibiotics had resulted in increased development and occurrence of bacterial resistance against commercially available antibiotics [21]. Therefore, the search for novel antibiotics of natural origins, particularly from marine resources [5, 15] and plants [7, 11] has been undertaken persistently. Nonetheless, studies which looked into the possibility of discovery of antimicrobial agent(s) from insects are scarce.

Insects, particularly the immature of flies, by nature of their biology and the ability to breed and thrive in septic habitats such as cadavers, carcasses, wounds and decaying organic matters, are infested with a great variety of microorganisms, some of which are pathogens [2]. In order to protect themselves from the undesirable effects of these microbes, insect immatures are known to produce and secrete potent anti-microbial substances [1, 4, 10, 16, 17] which may be the potential sources of novel antibiotics, especially for treatment of diseases caused by the multi-drug resistance strains such as the methicillin-resistant *Staphylococcus aureus* (MRSA).

The promising antibacterial activity of the local blowfly larvae, *Lucilia cuprina* against a wide range of pathogenic bacteria [19] which had in turn made it an effective medicinal larvae in maggot debridement therapy [13] as well as the ability of fly to thrive in septic environment have prompted our interest to screen the larval extract of other fly species against pathogenic bacteria. For screening purposes, it is essential to employ an in vitro antibacterial assay that is simple, reliable, sensitive, and most importantly, require a minimal amount of crude extract. The inability of turbidometric assay to differentiate dead and alive bacteria as well as the insensitivity of well-diffusion assay [9] had hindered the screening process.

The resazurin-based turbidometric (TB) assay was first used to quantify bacterial content in milk by Pesch and Simmert in 1929 [14]. Resazurin (7-Hydroxy-3H-phenoxazin-3-one 10-oxide) is a blue dye which can be irreversibly reduced to a pink and highly red fluorescent substance, resorufin by oxidoreductase within viable cells. The resorufin can be further reduced to a colorless and non-fluorescent molecule, hydroresorufin. In light of the simplicity and high-throughput of the resazurin-based TB assay, it has been employed in many studies as an antibacterial screening assay (Sarker et al., 2007; [18]; Hussain et al., 2011 [6]; Gahlaut & Chhillar, 2013; [3]), however, these studies only involved screening of antimicrobial activity of phytochemicals.

Therefore, the present study aimed to determine the antibacterial activity and properties (bactericidal or bacteriostatic) as well as minimum inhibitory concentrations (MICs) of larval extracts of *Lucilia cuprina, Sarcophaga peregrina,* and *Musca domestica* against *Staphylococcus aureus,* MRSA, *Pseudomonas aeruginosa* and *Escherichia coli* via resazurin-based TB assay, as well as to demonstrate the chemical profiles of these larval extracts using gas chromatography-mass spectrophotometry (GC-MS).

## Methods

### Larvae

The fly colonies of *Lucilia cuprina, Sarcophaga peregrina* and *Musca domestica* were maintained in the insectarium of Medical Entomology Unit, Institute for Medical Research (IMR), Kuala Lumpur under 12:12 h of light dark cycle at 22 ± 2 °C and 77.0 ± 2.53% humidity with continuous supply of water and granular sugar. Female flies were provided *with* raw cow liver (*L. cuprina*), raw cow lung (*S. peregrina*) or moistened mouse pellet (*M. domestica*) for oviposition. The resultant eggs were transferred onto fresh pieces of raw cow liver and mouse pellet (*L. cuprina*), raw cow lung (*S. peregrina*) or moistened mouse pellet (*M. domestica)* in clean containers and the hatched larvae were constantly supplied with fresh raw cow liver (*L.* cuprina) or raw cow lung (*S. peregrina*) and water for development into late second-instar larvae.

### Test bacteria

*Staphylococcus aureus* ATCC 25923*,* Methicillin-resistant *Staphylococcus aureus* (MRSA S914, a clinical isolate), *Pseudomonas aeruginosa* (ATCC 27853) and *Escherichia coli* (ATCC 25922) were kind gifts from the Bacteriology Unit, IMR, Kuala Lumpur. These bacterial cultures were maintained on blood agar (BA). All work pertaining to the handling of bacterial cultures were performed in a EuroClone® BioAir® Microbiological Safety Cabinet Class II type A2.

### Chemicals

All chemicals were purchased from Bio-Basic, Canada and Oxoid Ltd (BioFocus Saintifik Sdn.Bhd).

### Production of larval extract

The production of larval extract was performed according to the published protocols by Teh et al. [19] with slight modifications. Approximately 200 unsterile, 2 to 3 days-old fly larvae were collected from cow livers, cow lungs or moistened mouse pellet and transferred into a clean, disinfected 50 ml washing tube. The unsterile larvae were washed with 40 ml of 70% ethyl alcohol and rinsed three times with sterile distilled water. Washed larvae were blot-dried with sterile paper towels and transferred into another clean, disinfected 50 ml washing tube.

Larvae were homogenised with absolute methanol (200 larvae/ 100 ml methanol). The homogenate was then transferred into clean, disinfected 50 ml centrifuge tubes and centrifuged at 4000 x g for 30 min (Eppendorf® Centrifuge 5810R). The resultant yellowish supernatant was collected and transferred into clean, disinfected glass vials. Lastly, the supernatant was concentrated using a centrifugal vacuum concentrator (Genevac miVac Quattro Concentrator) to remove methanol. The vacuum-concentrated product, i.e., the larval extract was weighed before being kept at -70 °C. Prior to antibacterial assay, 200 mg larval extract was re-suspended in 1 ml sterile distilled water and filter-sterilised with Minisart® cellulose acetate membrane syringe filter with a pore size of 0.2 μm.

### Preparation of bacterial suspension

Bacteria stock cultures (*S. aureus,* MRSA*, P. aeruginosa* and *E. coli*) were sub-cultured onto BA plates and incubated overnight at 37 °C. The next day, three to four discrete bacterial colonies with similar morphology were inoculated into 10 ml sterile Mueller Hinton broth (MHB) and incubated overnight at 37 °C. The overnight bacterial suspensions were adjusted to 0.5 McFarland Standard with sterile MHB broth. To aid comparison, the adjustment of bacterial suspensions to the density of the 0.5 McFarland Standard was done against a white background with contrasting black lines.

### Preparation of resazurin solution

Resazurin solution was prepared by dissolving 337.5 mg of resazurin powder in 50 ml sterile distilled water in a disinfected beaker. A sterile vortex mixer was used to mix the solution for 1 h to ensure homogeneity. The preparation procedures were performed in dark and the resazurin solution was then kept in a brown bottle to prevent exposure to light since it is sensitive to light.

### Resazurin-based turbidometric assay and Minimum Inhibitory Concentration (MIC) determination

The resazurin-based turbidometric (TB) assay was adopted to demonstrate the inhibition effects of larval extract of *S. peregrina* and *M. domestica* against *S. aureus,* MRSA*, P. aeruginosa* and *E. coli.* The larval extract of *L. cuprina* was included into the study as another positive control in addition to standard antibiotics since its inhibitory effects had been demonstrated previously by Teh et al. [19] and therefore can validate and corroborate the feasibility of this assay. Broth microdilutions were performed precisely according to the Clinical and Laboratory Standards Institute (CLSI) protocol.

In a 96-well round-bottom microtiter plate, for each bacteria culture, the assay composed of one vertical row of broth sterility control, 3 vertical rows of larval extract sterility control (1 row each for *L. cuprina, S. peregrina* and *M. domestica*), 1 vertical row of growth control, 1 vertical row of antibiotic control and lastly, 3 vertical rows of larval extract test sample (1 row each for *L. cuprina, S. peregrina* and *M. domestica*). All eight wells in a vertical row were filled with 100 ul MHB. The first well of each vertical row contained 100 ul of sterile distilled water, larval extract of 200 mg ml^-1^, sterile distilled water, chloramphenicol (for *S. aureus* and MRSA) or gentamicin (for *P. aeruginosa* and *E.coli*) of 100 mg ml^-1^ and larval extract of 200 mg ml^-1^ for broth sterility control, larval extract sterility controls, growth control, antibiotic control and larval extract test samples, respectively. Subsequently, the mixture in the first well of each vertical row was mixed thoroughly. Then, a separate and sterile pipette was used to transfer 100 μl of mixture in the first well into the second well (2^-2^), and mixed thoroughly. Again, 100 μl of the mixture was transferred from the second well into the third well (2^-3^) and mixed thoroughly. This serial dilution was continued to the eighth well (2^-8^). Lastly, 100 μl was removed from the eighth well and discarded. The final concentration of antibiotics and larval extract was now one-half of the original concentration in each well.

Then, 5 μl of diluted bacterial suspension (1.5 x 10^6^ cell/ml) was added into all wells (except the broth sterility and larval extract sterility control column) and mixed thoroughly. Microdilution was performed in triplicates for each bacterial species. After an overnight incubation at 37 °C, 5 ul resazurin (6.75 mg ml^-1^) was added to all wells and incubated at 37 °C for another 4 h. Changes of color was observed and recorded. The lowest concentration prior to colour change was considered as the Minimum Inhibitory Concentration (MIC).

### Determination of antibacterial properties of larval extract

In order to elucidate the antibacterial properties (bactericidal or bacteriostatic) of larval extracts, a loopful of aliquots from the MIC wells was transferred onto brain heart infusion agar (BHIA) and incubated overnight. If bacteria failed to resume growth on BHIA after an overnight incubation, the larval extract was considered to be bactericidal, otherwise, it was bacteriostatic.

### Gas Chromatography-Mass Spectrometry (GC-MS)

To determine the chemical profile of larval extracts, 1.0 μl of methanol extract of *L. cuprina, S. peregrina* and *M. domestica* larvae (1 mg/ml) was injected into a gas chromatography system (Agilent 7890A) coupled with an inert mass spectrometer (Agilent 5975C) with triple-axis detector (quadrupole). The separation of larval extract was achieved using a DB5-MS UI capillary column (30 m x 0.25 mm x 0.25 μm; 5% polydimethylsiloxane) via an autosampler (CTC Analytics) in splitless mode. Helium was used as the carrier gas with a linear velocity of 1 ml/min. The injector temperature was set at 230 °C and oven temperature was kept at 70 °C for 2 min and then increased to 270 °C at 20 °C/min.

## Results

In the resazurin-based turbidometric (TB) assay, all sterility control wells for all tested bacteria remained as blue colour after an overnight incubation and followed by a 4-h incubation with resazurin. In contrast, all wells in the growth control column (contained growth medium and bacteria) of all tested bacteria had changed from blue to pink colour or from blue to pale pink (Additional files 1, 2, 3 and 4).

Table 1 demonstrated that the minimum inhibitory concentration (MIC) of the positive control, chloramphenicol against *S. aureus* was observed at the sixth well (2^-6^ = 1.56 mg ml^-1^) which was the last well prior to the occurrence of colour change. On the other hand, the MIC of the larval extract of *L. cuprina* was observed at the first well (2^-1^) which was equivalent to 100 mg ml^-1^. However, the larval extract of both *S. peregrina* and *M. domestica* were not active against *S. aureus* since all wells in both columns had changed from blue to pale pink, which indicated bacterial growth.

**Table 1:** MICs of standard antibiotics and larval extracts against bacteria

Bacteria	Antibiotics (100 mg ml^-1^)	Larval Extracts (200 mg ml^-1^)		
		*L. cuprina*	*S. peregrina*	*M. domestica*
*S. aureus*	Chloramphenicol 1.56	100.0	-	-
MRSA	Chloramphenicol 25.0	100.0	100.0	100.0
*P. aeruginosa*	Gentamicin 1.56	100.0	-	-
*E. coli*	Gentamicin 0.78	100.0	-	-

In contrast, the MIC of chloramphenicol against MRSA was as high as 25 mg ml^-1^ and all larval extracts (*L. cuprina, S. peregrina* and *M. domestica*) were inhibitory against MRSA at MICs of 100 mg ml^-1^. On the other hand, for the gram-negative bacteria, *P. aeruginosa* and *E. coli,* the MICs of gentamicin were 1.56 mg ml^-1^ and 0.78 mg ml^-1^, respectively. The larval extract of *L. cuprina* was active against both *P. aeruginosa* and *E. coli* at MIC of 100 mg ml^-1^, however, similar to those observed in *S. aureus,* larval extracts of *S. peregrina* and *M. domestica* did not exhibit any antibacterial activity against these gram-negative bacteria. The MICs of standard antibiotics and larval extracts against all tested bacteria were summarised in Table 1.

When aliquots were removed from the corresponding MIC wells of standard antibiotics (gentamicin or chloramphenicol) or *L. cuprina* larval extract, no bacterial growth was observed for all BHIA plates because bacteria cells were unable to resume growth (Additional file 5). However, aliquots of MRSA resumed growth on BHIA plates after it was removed from the MIC wells of *S. peregrina* and *M. domestica* larval extract (Additional file 6) though the resazurin-based TB assay demonstrating that the growth of MRSA was inhibited.

The GC-MS analysis had quantitatively identified as many as 20 organic compounds from the larval extract of *L. cuprina* and 17 of them were fatty acids or their derivatives (Table 2). Amongst these 20 compounds, α -Methyl-D-mannoside (18.41%) was found to be the most dominant compound, followed by hydroxypropyl ester of oleic acid (15.52%), oleic acid (8.20%), Methyl α-D-galactoside (7.25%) and palmitic acid (7.11%). The other compounds were present in trace amount (less than 5%). In contrast, only 5 compounds, of which all were methyl ester of fatty acids (palmitic acid, oleic acid, palmitoleic acid, myristic acid and linoleic acid) which were also present in the larval extract of *L. cuprina* were identified from the larval extracts of *S. peregrina* and *M. domestica* (Table 3).

**Table 2:** Chemical components of the methanol extract of larvae of *Lucilia cuprina*

Retention Time (Min)	Content (%)	Compound Name (NIST Library)	Chemical Formula/ Molecular Weight (g/mol)	Compound Nature
9.568	1.38	Phenol, 2,4-bis(1,1-dimethylethyl)-	C_14_H_22_O/ 206.32	Phenolic compound
10.621	7.25	Methyl α-D-galactopyranoside	C_7_H_14_O_6_/ 194.18	Glycoside
10.892	18.41	Methyl α-D-mannopyranoside	C_7_H_14_O_6_/ 194.18	Glycoside
12.118	0.84	Methyl tetradecanoate	C_15_H_30_O_2_/ 242.40	Fatty acid methyl ester (myristic acid methyl ester)
12.474	0.34	Tetradecanoic acid	C_14_H_28_O_2_/ 228.37	Fatty acid (myristic acid)
14.035	2.64	9-hexadecenoic acid, methyl ester	C_17_H_32_O_2_/ 268.43	Fatty acid methyl ester (palmitoleic acid methyl ester)
14.246	4.47	Hexadecanoic acid, methyl ester	C_17_H_34_O_2_/ 270.45	Fatty acid methyl ester (palmitic acid methyl ester)
14.322	0.39	Benzenepropanoic acid, 3,5-bis (1,1-dimethyethyl)-4-hydroxy-, methyl ester	C_18_H_28_O_3_/ 292.41	Aromatic acid ester
14.403	2.81	Cis-9-hexadecenoic acid	C_16_H_30_O_2_/ 254.41	Fatty acid (isomer of palmitoleic acid)
14.608	7.11	n-Hexadecanoic acid	C_16_H_32_O_2_/ 256.42	Fatty acid (palmitic acid)
15.894	2.13	9,12-octadecadienoic acid (z,z)-, methyl ester	C_19_H_34_O_2_/ 294.47	Fatty acid methyl ester (linoleic acid methyl ester)
15.959	3.91	9-octadecenoic acid (z)-, methyl ester	C_19_H_36_O_2_/ 296.49	Fatty acid methyl ester (oleic acid methyl ester)
16.256	1.53	9,12-octadecadienoic acid (z,z)-	C_18_H_32_O_2_/ 280.45	Fatty acid (linoleic acid)
16.320	8.20	Oleic acid	C_18_H_34_O_2_/ 282.46	Fatty acid
16.515	1.55	Cis-vaccenic acid	C_18_H_34_O_2_/ 282.46	Fatty acid (isomer of oleic acid)
17.401	0.19	5,8,11,14-eicosatetraenoic acid, methyl ester (all-z)-	C_21_H_34_O_2_/ 318.49	Fatty acid methyl ester (arachidonic acid methyl ester)
19.594	3.93	Hexadecanoic acid, 2-hydroxy-1-(hydroxymethyl) ethyl ester	C_19_H_38_O_4_/ 330.50	Fatty acid ethyl ester of glycerol (Palmitic acid β-monoglyceride)
19.762	0.72	1,2-benzenedicarboxylic acid, mono(2-ethylhexyl) ester	C_16_H_22_O_4_/ 278.34	Benzoic acid
21.182	15.52	Oleic acid, 3-hydroxypropyl ester	C_21_H_40_O_3_/ 340.54	Fatty acid ester
21.388	2.83	Octadecanoic acid, 2,3-dihydroxypropyl ester	C_21_H_42_O_4_/ 358.56	Fatty acid ester of glycerol (Stearic acid α-monoglyceride)

**Table 3:** Chemical components of the methanol extract of larvae of *Sarcophaga peregrina* and *Musca domestica*

Retention Time (Min)	Content (%)	Compound Name (NIST Library)	Chemical Formula/ Molecular Weight (g/mol)	Compound Nature
12.115	4.05	Methyl tetradecanoate	C_15_H_30_O_2_/ 242.40	Fatty acid methyl ester (myristic acid methyl ester)
14.038	8.36	9-hexadecenoic acid, methyl ester	C_17_H_32_O_2_/ 268.43	Fatty acid methyl ester (palmitoleic acid methyl ester)
14.249	36.78	Hexadecanoic acid, methyl ester	C_17_H_34_O_2_/ 270.45	Fatty acid methyl ester (palmitic acid methyl ester)
15.891	2.96	9,12-octadecadienoic acid (z,z)-	C_18_H_32_O_2_/ 280.45	Fatty acid (linoleic acid)
15.956	10.29	9-octadecenoic acid (z)-, methyl ester	C_19_H_36_O_2_/ 296.49	Fatty acid methyl ester (oleic acid methyl ester)

## Discussion

In resazurin-based TB assay, viable and metabolically active bacteria cells irreversibly reduced the blue dye, resazurin to a pink and highly red fluorescent compound, resorufin and finally to a colourless and non-fluorescent molecule, hydroresorufin by oxidoreductase [8]. Such change of colour can be observed visually and therefore spectrophotometer is not needed in this assay as compared to the conventional TB assay.

Generally, the larval extract of *L. cuprina* was inhibitory against all tested bacteria, whilst the larval extract of *S. peregrina* and *M. domestica* were only active against MRSA. The broad spectrum inhibitory effects of the larval extract of *L. cuprina* against all tested bacteria in the resazurin-based TB assay were in agreement with those reported by Teh et al. [19] who employed conventional TB assay, and this again substantiated that the resazurin-based TB assay generated comparable results with the conventional TB assay and therefore can be considered as a simple, reliable and feasible antibacterial assay since it does not require a spectrophotometer to determine the bacterial growth. On the other hand, it was noteworthy that the MICs of *L. cuprina* larval extract as determined in the present study (100 mg ml^-1^) was relatively higher than those reported by Teh et al. [19]. The inconsistency in the MICs value though using the same fly species (*L. cuprina)* could be due to different definitions of MIC. Teh et al. [19] defined the MIC endpoints of larval extract against bacteria as the lowest concentration of larval extract (mg ml^-1^) resulting in at least 50% bacterial growth inhibition relative to that of the corresponding controls. In contrast, the present study defined MIC as the lowest concentration of larval extract resulting in color change (from blue to pink) or resazurin reduction. Since reduction of resazurin could only performed by viable bacterial cells, therefore, the MICs determined by the resazurin-based TB assay were relatively higher as compared to those determined via conventional TB assay [19] because more larval extract was required to inhibit bacterial growth to less than 80 bacterial cells ([12] had demonstrated that visible change of color from blue to pink can be detected in as few as 80 cells). Besides, the use of heavier bacterial inoculum in the present study i.e. 1.5 x 10^6^ colony-forming unit/ ml (CFU/ml) compared to only 1.0 x 10^2^ CFU/ml in the previous study by Teh et al. [19] may also attribute to the discrepancy in MICs.

To the best of the author’s knowledge, the inhibitory effect as well as the MICs of the *S. peregrina* and *M. domestica* larval extract against MRSA had never been determined. The apparent potency of the *L. cuprina, S. peregrina* and *M. domestica* larval extract against MRSA provided promising input for probable identification, isolation and purification of novel effective antibacterial compound(s) of natural origin, particularly to combat the dreadful bacterial strain, MRSA.

On the other hand, the inactivity of *M. domestica* larval extract against *S. aureus, P. aeruginosa* and *E. coli* was not in agreement with those reported by other investigators. Golebiowski et al. [4] who had successfully identified 7 compounds from the larvae of *M. domestica* had revealed that 2,4-decadienal exhibited the strongest antibacterial activity among the other 6 compounds, with MIC of 64 mg ml^-1^ against *S. aureus* and 512 mg ml^-1^ against *P. aeruginosa* and *E. coli*. The discrepancies between the present study and those reported by Golebiowski et al. [4] could be partly due to a lower bacterial inoculum tested (5 x 10^5^ CFU/ml) and also the employment of different antibacterial assay [turbidometric assay in the study by Golebiowski et al. [4]. In the study by Golebiowski et al. [4], the MICs of active compounds were defined as the lowest concentration of active compound at which growth inhibition was clearly visible (absence of turbidity and a pellet at the bottom of the well). Such MIC determination method could result in overestimating the inhibitory effects of the active compounds since visual absence of turbidity and a pellet at the bottom of the well may not guarantee bacterial inhibition as bacterial growth can still occur microscopically. On the other hand, for resazurin-based TB assay, the reduction of resazurin to resorufin by viable cells which in turn lead to visible change of color from blue to pink can be detected in as few as 80 cells [12] and therefore reduced the likelihood of overestimating the inhibitory effect of larval extract.

In terms of antibacterial properties, the inability of bacterial cells to resume growth on the brain heart infusion agar (BHIA) after being transferred from the MIC wells indicated that the standard antibiotics (chloramphenicol for *S. aureus* and MRSA; gentamicin for *P. aeruginosa* and *E.coli*) and *L. cuprina* larval extract were indeed bactericidal against all tested bacteria at the corresponding MICs. On the other hand, larval extracts of *S. peregrina* and *M. domestica* larval extract were unable to suppress the growth of MRSA on BHIA and this signposted that the larval extracts of *S. peregrina* and *M. domestica* exerted bacteriostatic effect against MRSA. Although larval extracts exhibited different properties of antibacterial activity (bactericidal or bacteriostatic) against bacteria, the clinical importance of bacteriostatic versus bactericidal effect on microorganisms is under dispute. Therefore, when screening potential substances for antibacterial activity, the antibacterial properties (bactericidal or bacteriostatic) of that particular substance should never be used to rule out its potential value as an efficient antibacterial drug. It should also be noted that although the MICs of larval extract were higher than the MICs of standard antibiotics, the standard antibiotics were composed of purified active ingredients as compared to the crude extracts of fly larvae. Therefore, a smaller amount of larval extract is expected to exhibit the antibacterial activity if the purified form of larval extract could be produced which would serve as the leads for synthesis of novel antimicrobial products of natural origin.

The chemical analysis of larval extracts revealed that fatty acids were the dominant compounds. Fatty acids had been reported to inhibit bacterial growth by disruption of bacterial membranes or inhibition of fatty acid synthesis [20]. Zheng et al. [22] reported that long chain unsaturated fatty acids such as oleic acid, linoleic acid, palmitoleic acid and arachidonic acid inhibited bacterial growth (*S. aureus*) by inhibiting the bacterial enoyl-acyl carrier protein reductase (FabI), which is an essential components of bacterial fatty acid synthesis. Therefore, it is not surprising that the antibacterial activities of larval extracts were contributed by the combination of fatty acids. This may partly explains the apparent potency of *L. cuprina* larval extract as compared to the larval extracts of *S. peregrina* and *M. domestica* since it contained more fatty acids. Nonetheless, one should underscore that these preliminary GC-MS analysis only demonstrated the chemical profile of non-volatile compounds from the larval extracts, in order not to overlook the other volatile compounds which may have antibacterial activities, future work to derivatise the larval extract using silylating reagent such as N,O-bis(trimethylsilyl) trifluoroacetamide (BSTFA) should be undertaken for potential identification and isolation of novel antimicrobial substance(s). Once these compounds have been identified, their antibacterial activity will be tested, both singly and in combinations in the future studies. Subsequently, the cytotoxicity of these compounds on mammalian cell lines will also be assessed to warrant safe use of these compounds in human.

## Conclusions

In short, the resazurin-based turbidometric assay is a simple, reliable and feasible screening assay in assessing the antibacterial activity of larval extract of flies. This assay evidently demonstrated the antibacterial activity of *L. cuprina, S. peregrina* and *M. domestica* larval extracts against MRSA, with *L. cuprina* exerted the broadest antibacterial activity against both gram-positive (*S. aureus* and MRSA) and gram-negative bacteria (*P. aeruginosa* and *E. coli*). The present study also revealed a potential room for the development of novel and effective natural disinfectant(s) and antibacterial agent(s) from flies. Further work of derivatisation and characterization of the larval extract samples to retrieve other non-volatile compounds is greatly warranted to produce a detailed chemical profile as an informative guidance for subsequent identification of antibacterial compound(s). In addition, additional work to screen more fly species using resazurin-based TB assay for screening of antimicrobial activity for probable identification and isolation of potential antimicrobial substances should also be undertaken. If successful, these isolated and purified active substances may then be used as an alternative for maggot debridement therapy for entomo-phobia patients as well as in combating the increasing threat of emergence of multidrug resistance bacterial strains, particularly the MRSA.

## Additional file

Additional file 1:
*S. aureus* microtiter plate after an overnight incubation and addition of resazurin dye (B = broth sterility control; LC = *L. cuprina* larval extract sterility control; SP = *S. peregrina* larval extract sterility control and MD = *M. domestica* larval extract sterility control; G = growth control; Ab = chloramphenicol; yellow circle indicates the MIC). (EPS 13.9 mb)
Additional file 2: MRSA microtiter plate after an overnight incubation and addition of resazurin dye (B = broth sterility control; LC = *L. cuprina* larval extract sterility control; SP = *S. peregrina* larval extract sterility control and MD = *M. domestica* larval extract sterility control; G = growth control; Ab = chloramphenicol; yellow circle indicates the MIC). (EPS 13 mb)
Additional file 3:
*P. aeruginosa* microtiter plate after an overnight incubation and addition of resazurin dye (B = broth sterility control; LC = *L. cuprina* larval extract sterility control; SP = *S. peregrina* larval extract sterility control and MD = *M. domestica* larval extract sterility control; G = growth control; Ab = gentamicin; yellow circle indicates the MIC). (EPS 14.3 mb)
Additional file 4:
*E. coli* microtiter plate after an overnight incubation and addition of resazurin dye (B = broth sterility control; LC = *L. cuprina* larval extract sterility control; SP = *S. peregrina* larval extract sterility control and MD = *M. domestica* larval extract sterility control; G = growth control; Ab = gentamicin; yellow circle indicates the MIC). (EPS 16.9 mb)
Additional file 5: Bactericidal effects of standard antibiotics and *L. cuprina* larval extract against all tested bacteria. The left column of BHIA plates were inoculated with aliquots from the MIC wells of the corresponding standard antibiotics (gentamicin or chloramphenicol) for each tested bacteria (*P. aeruginosa, E. coli, S. aureus* and MRSA) whilst the right column of BHIA plates were inoculated with aliquots from the MIC wells of *L. cuprina* larval extract for each tested bacteria (*S. aureus,* MRSA, *P. aeruginosa* and *E. coli*). (EPS 8.88 mb)
Additional file 6: Apparent potency of *L. cuprina* larval extract against MRSA. The brain-heart infusion agar (BHIA) plate was inoculated with a loop-full of aliquots from the MIC wells of *L. cuprina* (upper left)*, S. peregrina* (upper right) and *M. domestica* (middle bottom) larval extract against MRSA after an overnight incubation. (EPS 8.67 mb)

## Abbreviations

BSTFA: N,O-bis(trimethylsilyl) trifluoroacetamide
CLSI: Clinical and Laboratory Standards Institute
FAMEs: Fatty acid methyl esters
GC-MS: Gas chromatography mass spectrometry
IMR: Institute for Medical Research
MIC: Minimum Inhibitory Concentration
MRSA: methicillin-resistant *Staphylococcus aureus*
TB: Turbidometric assay
